# Assessment of subclinical LV myocardial dysfunction in T2DM patients with diabetic peripheral neuropathy: a cardiovascular magnetic resonance study

**DOI:** 10.1186/s12933-024-02307-x

**Published:** 2024-06-24

**Authors:** Xue-Ming Li, Ke Shi, Li Jiang, Jing Wang, Wei-Feng Yan, Yue Gao, Meng-Ting Shen, Rui Shi, Ge Zhang, Xiao-Jing Liu, Ying-Kun Guo, Zhi-Gang Yang

**Affiliations:** 1 Department of Radiology, West China Hospital, Sichuan University, 37# Guo Xue Xiang, Chengdu, Sichuan China; 2 Laboratory of Cardiovascular Diseases, Regenerative Medicine Research Center, West China Hospital, Sichuan University, 37# Guo Xue Xiang, Chengdu, Sichuan China; 3 Key Laboratory of Birth Defects and Related Diseases of Women and Children of Ministry of Education, Department of Radiology, West China Second University Hospital, Sichuan University, 20# South Renmin Road, Chengdu, Sichuan China

**Keywords:** Type 2 diabetes mellitus, Diabetic peripheral neuropathy, Strain, Magnetic resonance imaging, Left ventricle

## Abstract

**Background:**

Diabetic peripheral neuropathy (DPN) is the most prevalent complication of diabetes, and has been demonstrated to be independently associated with cardiovascular events and mortality. This aim of this study was to investigate the subclinical left ventricular (LV) myocardial dysfunction in type 2 diabetes mellitus (T2DM) patients with and without DPN.

**Methods:**

One hundred and thirty T2DM patients without DPN, 61 patients with DPN and 65 age and sex-matched controls who underwent cardiovascular magnetic resonance (CMR) imaging were included, all subjects had no symptoms of heart failure and LV ejection fraction ≥ 50%. LV myocardial non-infarct late gadolinium enhancement (LGE) was determined. LV global strains, including radial, circumferential and longitudinal peak strain (PS) and peak systolic and diastolic strain rates (PSSR and PDSR, respectively), were evaluated using CMR feature tracking and compared among the three groups. Multivariable linear regression analyses were performed to determine the independent factors of reduced LV global myocardial strains in T2DM patients.

**Results:**

The prevalence of non-infarct LGE was higher in patients with DPN than those without DPN (37.7% vs. 19.2%, p = 0.008). The LV radial and longitudinal PS (radial: 36.60 ± 7.24% vs. 33.57 ± 7.30% vs. 30.72 ± 8.68%; longitudinal: − 15.03 ± 2.52% vs. − 13.39 ± 2.48% vs. − 11.89 ± 3.02%), as well as longitudinal PDSR [0.89 (0.76, 1.05) 1/s vs. 0.80 (0.71, 0.93) 1/s vs. 0.77 (0.63, 0.87) 1/s] were decreased significantly from controls through T2DM patients without DPN to patients with DPN (all p < 0.001). LV radial and circumferential PDSR, as well as circumferential PS were reduced in both patient groups (all p < 0.05), but were not different between the two groups (all p > 0.05). Radial and longitudinal PSSR were decreased in patients with DPN (p = 0.006 and 0.003, respectively) but preserved in those without DPN (all p > 0.05). Multivariable linear regression analyses adjusting for confounders demonstrated that DPN was independently associated with LV radial and longitudinal PS (β = − 3.025 and 1.187, p = 0.014 and 0.003, respectively) and PDSR (β = 0.283 and − 0.086, p = 0.016 and 0.001, respectively), as well as radial PSSR (β = − 0.266, p = 0.007).

**Conclusions:**

There was more severe subclinical LV dysfunction in T2DM patients complicated with DPN than those without DPN, suggesting further prospective study with more active intervention in this cohort of patients**.**

## Background

The substantial global increase in the incidence of diabetes has led to a parallel increase in the rates of diabetes-related deaths and complications [1, 2]. Diabetic peripheral neuropathy (DPN) involving the outer nerves of the limbs is one of the most common complications of diabetes and affects over 50% of patients with type 2 diabetes mellitus (T2DM) [3]. In addition, it has been demonstrated to be independently associated with cardiovascular events and mortality in a number of studies [4–6]. Therefore, early detection of myocardial impairment in this cohort of patients is essential to prevent progression and subsequent increases in morbidity and mortality.

Current screening methods for diabetic cardiomyopathy mainly rely on left ventricular ejection fraction (LVEF) measurements that are based on global ventricular volume measurements, whether using echocardiography or CMR, which have inherent limitations, as they can only detect moderate to severe cardiac dysfunction. Interestingly, a large body of published data has shown that the echocardiography speckle tracking and cardiovascular magnetic resonance feature tracking (CMR-FT) techniques can detect and monitor the progression of subclinical myocardial dysfunction, which can further predict cardiovascular events [7, 8]. Although echocardiography is currently the most convenient method for cardiac examination, it has a low spatial resolution and is highly dependent on the operator and angle, making it unsuitable for some patients with a poor echo window. CMR-FT, which is derived from a cine balanced steady-state free precession (bSSFP) sequence, has the advantages of a wide field of view, no anatomical plane restriction, and a semiautomatic and time-saving postprocessing procedure. In addition, late gadolinium enhancement (LGE) is a most advantage of MRI compared to the echocardiography, which is used to assess myocardial tissue characteristics [9, 10].

Therefore, the aim of this study was to evaluate subclinical left ventricular (LV) myocardial dysfunction in type 2 diabetes mellitus (T2DM) patients with and without DPN using CMR-FT. The results might provide additional information on the link between the risk of cardiovascular disease and DPN.

## Methods

### Study population

Between January 2015 and June 2023, T2DM patients who had undergone CMR examinations were initially screened. T2DM was diagnosed according to the current American Diabetes Association guideline [11]. DPN was clinically diagnosed using the diagnostic criteria introduced by the American Diabetes Association in 2017 [12]. Chronic kidney disease (CKD) is clinically defined by the presence of persistent estimated glomerular filtration rate (eGFR) < 60 mL/min/1.73 m^2^. The exclusion criteria included patients with coronary artery disease (confirmed by electrocardiogram, echocardiography, angiography, coronary computed tomographic angiography or CMR, or previous myocardial infarction or coronary revascularization), symptoms of heart failure, left ventricular ejection fraction < 50% on echocardiography or CMR imaging, other primary cardiomyopathies, moderate to severe valvular disease, atrial fibrillation, severe renal failure (eGFR < 30 mL/min/1.73 m^2^), other causes of peripheral neuropathy (including chronic inflammatory demyelinating polyradiculoneuropathy, mononeuropathy, or conditions caused by vitamin B deficiency and thyroid dysfunction), incomplete clinical records and poor CMR image quality inadequate for analysis. Finally, 191 patients with T2DM were enrolled in this study, including 130 patients without DPN (75 males and 55 females, mean age 56.5 ± 9.7 years) and 61 patients with DPN (38 males and 23 females, mean age 55.3 ± 10.2). In addition, 65 age- and sex-matched healthy individuals (34 males and 31 females; mean age, 55.4 ± 9.9 years) were enrolled as the control group. The inclusion criteria for the control group were as follows: no diabetes or impaired glucose tolerance, hypertension, ischemic heart disease, cardiomyopathy, abnormal electrocardiogram, abnormalities detected with CMR (abnormal ventricular motion, valvular stenosis or regurgitation, decreased LVEF, etc.) or other cardiovascular disease-related symptoms.

This study (No. 2019-878) was approved by the Biomedical Research Ethics Committees of our hospital and complied with the Declaration of Helsinki. Written informed consent was waived due to the retrospective nature of the study.

### CMR protocol

All subjects underwent CMR imaging on a 3 T whole-body scanner MAGNETOM Skyra or Trio Tim (Siemens Medical Solutions, Erlangen, Germany) in the supine position. The balanced steady-state free precession sequence (repetition time [TR] = 3.4 ms or 2.81 ms, echo time [TE] = 1.22 ms, flip angle = 50° or 40°, slice thickness = 8 mm, field of view [FOV] = 340 × 285 mm or 250 × 300 mm, matrix size = 256 × 166 or 208 × 139) with breath holding and ECG triggering was performed to acquire cine images, including a stack of contiguous short-axis slices covering the entire left ventricle from base to apex and one four- and two-chamber long-axis slice. Twenty-five frames were reconstructed per breath-hold acquisition. The LGE images in the entire LV short-axis stack and from the two-, three- and four-chamber views were acquired to exclude patients with infarct LGE and identify those with non-infarct LGE 10–15 min after contrast agent administration using the segmented-turbo-FLASH–phase-sensitive inversion recovery sequence (TR/TE, 300 ms/1.44 ms or 750 ms/1.18 ms, slice thickness, 8 mm, FOV, 275 × 400 mm or 400 × 270 mm, matrix size = 256 × 184 and flip angle, 40°).

### Image analysis

All CMR data were uploaded to offline commercial software (Cvi42, v.5.11.2; Circle Cardiovascular Imaging, Inc., Calgary, Canada) and analyzed by two experienced radiologists with more than five years of experience in CMR interpretation, who were blinded to the clinical data.

The endo- and epicardial contours of left ventricle were semiautomatically delineated at the end-diastolic and end-systolic phases on the short-axis cine images in the Short-3D module. The papillary muscles and moderator bands were included in the ventricular cavity and excluded from the myocardial muscle. The global parameters of LV geometry and function, including end-diastolic volume (EDV), end-systolic volume (ESV), stroke volume (SV), LVEF and LV mass, were computed automatically; LVEDV, LVESV, LVSV and LV mass were indexed to the body surface area (BSA) (LVEDVI, LVESVI, LVSVI and LVMI, respectively). The LV remodeling index was calculated as the ratio of LV mass to LVEDV.

**Fig. 1:**
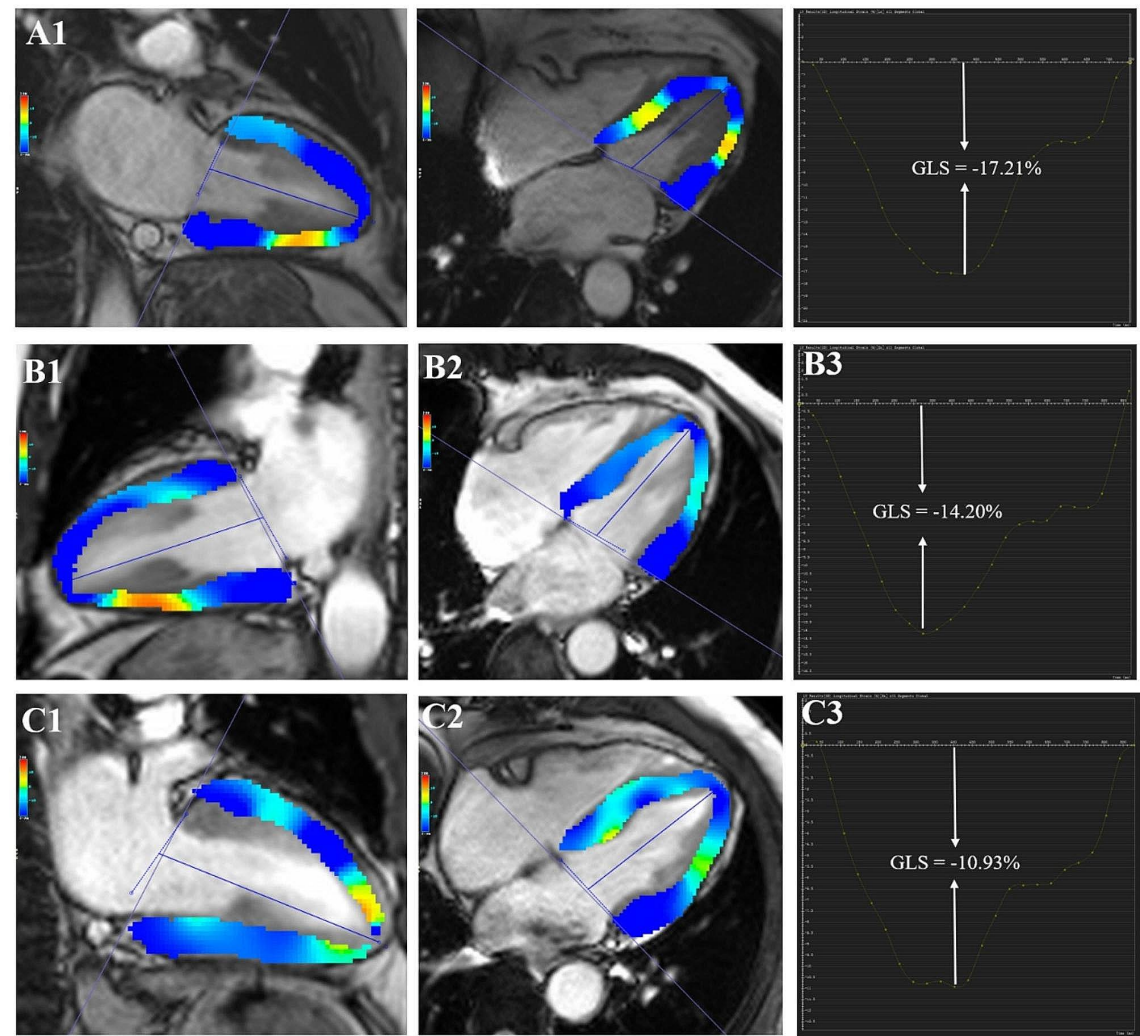
Representative CMR left ventricular pseudocolor images of long-axis two- and four-chamber images at the end-systole and CMR-derived global longitudinal peak strain (GLS) curves in a normal control (**A1**–**A3**), T2DM patient without DPN (**B1**–**B3**) and T2DM patient complicated with DPN (**C1**–**C3**)

The short-axis and long-axis four- and two-chamber cine images were loaded into the tissue tracking module for the LV myocardial strain analysis. The endo- and epicardial contours were semiautomatically delineated with papillary muscles and moderator bands excluded in all series at the end-diastolic phase. Subsequently, the LV global strain parameters (Fig. 1) were acquired automatically, including global radial, circumferential and longitudinal peak strain (PS) and peak systolic and diastolic strain rates (PSSR and PDSR, respectively). PS was defined as the relative thickening, shortening and lengthening of the myocardium from end diastole (reference phase). PSSR and PDSR are defined as the maximum strain rate during the contraction and relaxation phases, respectively.

For late gadolinium enhancement (LGE) analysis, the LGE images were visually evaluated by two observers in combination and categorized into 3 patterns, that is none, infarct, or non-infarct patterns [13].

### Statistical analysis

All continuous variables were assessed for normality with the Shapiro–Wilk test. Continuous variables are presented as the means ± standard deviations (SD) for normally distributed variables or the medians (25–75% interquartile ranges) for skewed variables. Comparisons between three groups were performed with one-way analysis of variance (ANOVA) with the post hoc Bonferroni correction for normally distributed variables and Kruskal–Wallis tests for variables with skewed distributions. The duration of T2DM and HbA1c levels were compared between the patient groups with Mann–Whitney *U* test. Categorical variables are presented as percentages and were compared using Chi-square tests. Variables with p < 0.1 in the univariable analysis, as well as age, sex and diabetes duration were included in the stepwise multivariable linear regression analyses to determine the predictors of LV systolic and diastolic function in patients with T2DM. The diabetic duration was divided into long (> 5 years) and short (≤ 5 years) term duration which were included in the univariable and multivariable analysis. Two-tailed p values < 0.05 were considered statistically significant, and statistical analyses were performed with SPSS version 23.0 (IBM, Armonk, New York, USA).

## Results

### Baseline characteristics

The main clinical characteristics of the study cohort are shown in Table 1. Although age was not significantly different between the patient groups, patients with DPN had a longer diabetes duration (p = 0.003) and higher incidences of dyslipidemia (63.9% vs. 42.3%, p = 0.008), retinopathy (29.5% vs. 4.6%, p < 0.001) and CKD (24.6% vs. 12.3%, p = 0.037) than those without DPN. As expected, both patient groups showed higher fasting blood glucose levels than the control group (all p < 0.001), and patients with DPN had significantly higher HbA1c levels than those without DPN (p = 0.001). In addition, BMI, mean SBP and DBP were significantly higher in patients with or without DPN than in controls (all p < 0.05), but they were not significantly different between the patient groups.

**Table 1:** Baseline characteristics of the study cohort

	Controls (n = 65)	T2DM (DPN−) (n = 130)	T2DM (DPN+) (n = 61)
Male, n (%)	34 (52.3)	75 (57.7)	38 (62.3)
Age (years)	55.4 ± 9.9	56.5 ± 9.7	55.3 ± 10.2
BMI (kg/m^2^)	22.81 ± 2.38	24.67 ± 3.48*	24.45 ± 3.39*
BSA (kg/m^2^)	1.72 ± 0.19	1.72 ± 0.16	1.72 ± 0.17
Rest heart rate (beats/min)	71.7 ± 11.3	74.6 ± 11.9	75.9 ± 10.2
Systolic blood pressure (mmHg)	117.3 ± 13.4	127.0 (118.0, 140.0)*	130.0 (120.0, 144.5)*
Diastolic blood pressure (mmHg)	75.0 (70.0, 98.0)	80.0 (73.0, 86.5)*	80.0 (74.0, 85.0)*
Hypertension, n (%)	NA	65 (50.0)	32 (52.5)
Diabetes duration (years)	NA	4.5 (2, 10)	9 (3, 14)^†^
Dyslipidemia, n (%)	NA	55 (42.3)	39 (63.9)^†^
Retinopathy, n (%)	NA	6 (4.6)	18 (29.5)^†^
CKD, n (%)	NA	16 (12.3)	15 (24.6)^†^
Laboratory data			
FBG (mmol/L)	5.12 (4.78, 5.73)	7.30 (6.10, 8.95)*	8.32 (6.44, 11.78)*
HbA1c (%)	NA	6.8 (6.2, 7.7)	7.7 (6.7, 9.4)^†^
Triglycerides (mmol/L)	1.25 (0.99, 1.84)	1.31 (0.91, 1.95)	1.39 (1.01, 2.15)
Total cholesterol (mmol/L)	4.54 ± 0.80	4.32 ± 0.97	4.19 (3.66, 5.35)
HDL (mmol/L)	1.31 (1.09, 1.56)	1.17 (0.99, 1.48)	1.20 (0.91, 1.44)
LDL (mmol/L)	2.67 ± 0.70	2.42 (1.86, 3.03)	2.44 (1.85, 3.44)
eGFR (mL/min/1.73 m^2^)	92.11 ± 18.62	96.0 (79.7, 102.9)	98.9 (84.4, 109.0)
Medications, n (%)			
Statin	NA	29 (22.3)	26 (42.6)^†^
Biguanides	NA	75 (57.7)	37 (60.7)
Sulfonylureas	NA	31 (23.8)	16 (26.2)
α-Glucosidase inhibitor	NA	48 (36.9)	25 (41.0)
GLP-1/DDP-4 inhibitor	NA	13 (10.0)	8 (13.1)
SGLT2	NA	3 (2.3)	4 (6.6)
Insulin	NA	28 (21.5)	36 (59.0)^†^
ACEI/ARB	NA	33 (25.4)	11 (19.7)
β-blocker	NA	12 (9.3)	5 (8.2)
Calcium channel blocker	NA	35 (26.9)	17 (27.9)

The values are mean ± SD, Numbers in the brackets are percentages

*T2DM* type 2 diabetes mellitus, *DPN* diabetic peripheral neuropathy, *BMI* body mass index, *BSA* body surface area, *CKD* chronic kidney disease, *FBG* fasting blood glucose, *HbA1c* glycated hemoglobin, *HDL* high-density lipoprotein cholesterol, *LDL* low-density lipoprotein cholesterol, *eGFR* estimated glomerular filtration rate, *ACEI* angiotensin converting enzyme inhibitor, *ARB* angiotensin II receptor blocker, *GLP-1* glucagon-like peptide-1, *DPP-4* dipeptidyl peptidase-4, *SGLT2* sodium-dependent glucose transporters 2

*p < 0.05 vs. controls

^†^p < 0.05 vs. T2DM (DPN−) group

No significant differences in the use of medications were observed between patient groups except for statins and insulin, which were most frequently used in patients with DPN (p = 0.006 and < 0.001, respectively).

### Characteristics of LV geometry and strain parameters

The CMR findings for the study cohort are shown in Table 2. T2DM patients without and with DPN had a larger LV mass than the control group (p = 0.007 and 0.002, respectively), and the differences were present even after adjustment for BSA (p = 0.006 and 0.001, respectively). The LV remodeling index in the patient groups without and with DPN was significantly higher than that in the control group (p = 0.034 and = 0.017 respectively). Besides, the prevalence of non-infarct LGE was significantly higher in subjects with DPN compared with those without DPN (37.7% vs. 19.2%, p = 0.008). The LVEDV, LVEDVI, LVESV, LVESVI, LVSV, LVSVI and LVEF were not significantly different among the three groups (all p > 0.05).

**Table 2:** Comparison of CMR findings among T2DM patients with/without DPN and normal controls

	Controls	T2DM (DPN−)	T2DM (DPN+)
LV Geometry			
LVEDV (mL)	123.93 ± 22.73	128.43 ± 29.02	131.16 ± 34.47
LVEDVI (ml/m^2^)	72.12 ± 11.29	74.71 ± 15.93	75.59 ± 18.13
LVESV (mL)	43.96 ± 11.97	47.62 ± 16.44	47.97 ± 18.08
LVESVI (ml/m^2^)	25.53 ± 6.47	27.31 ± 9.14	27.54 ± 10.03
LVSV (mL)	79.97 ± 15.78	81.29 ± 18.46	82.99 ± 21.87
LVSVI (ml/m^2^)	46.59 ± 8.36	47.39 ± 10.11	48.06 ± 11.56
LVEF (%)	64.60 ± 6.29	63.89 ± 6.99	63.86 ± 7.17
LV mass (g)	72.59 ± 18.70	83.16 ± 23.64*	86.54 ± 24.47*
LVMI (g/m^2^)	42.33 ± 9.02	48.06 ± 12.40*	49.92 ± 12.48*
LVRI (g/mL)	0.59 ± 0.13	0.66 ± 0.18*	0.68 ± 0.17*
Non‑infarct LGE, n (%)	NA	25 (19.2)	23 (37.7)^†^
LV Strains			
PS, %			
Radial	36.60 ± 7.24	33.57 ± 7.30*	30.72 ± 8.68*^†^
Circumferential	− 20.97 ± 2.59	− 20.01 ± 2.40*	− 19.15 ± 2.99*
Longitudinal	− 15.03 ± 2.52	− 13.39 ± 2.48*	− 11.89 ± 3.02*^†^
PSSR, 1/s			
Radial	2.06 (1.68, 2.36)	1.75 (1.54, 2.34)	1.64 (1.41, 2.19)*
Circumferential	− 1.06 ± 0.20	− 1.05 ± 0.20	− 1.00 ± 0.19
Longitudinal	− 0.79 (− 0.92, − 0.72)	− 0.75 (− 0.88, − 0.64)	− 0.72 (− 0.82, − 0.64)*
PDSR, 1/s			
Radial	− 2.36 (− 3.15, − 2.01)	− 2.09 (− 2.74, − 1.77)*	− 1.93 (− 2.41, − 1.56)*
Circumferential	1.26 ± 0.28	1.15 ± 0.24*	1.13 ± 0.27*
Longitudinal	0.89 (0.76, 1.05)	0.80 (0.71, 0.93)*	0.77 (0.63, 0.87)*^†^

The values are mean ± SD, Numbers in the brackets are percentages

*T2DM* type 2 diabetes mellitus, *DPN* diabetic peripheral neuropathy, *LV* left ventricular, *EDV* end diastolic volume, *ESV* end systolic volume, *SV* stroke volume, *EF* ejection fraction, *LVMI* LV mass index, *I* indexed to BSA, *PS* peak strain, *PSSR* peak systolic strain rate, *PDSR* peak diastolic strain rate

*p < 0.05 vs. controls

^†^p < 0.05 vs. T2DM (DPN−) group

**Fig. 2:**
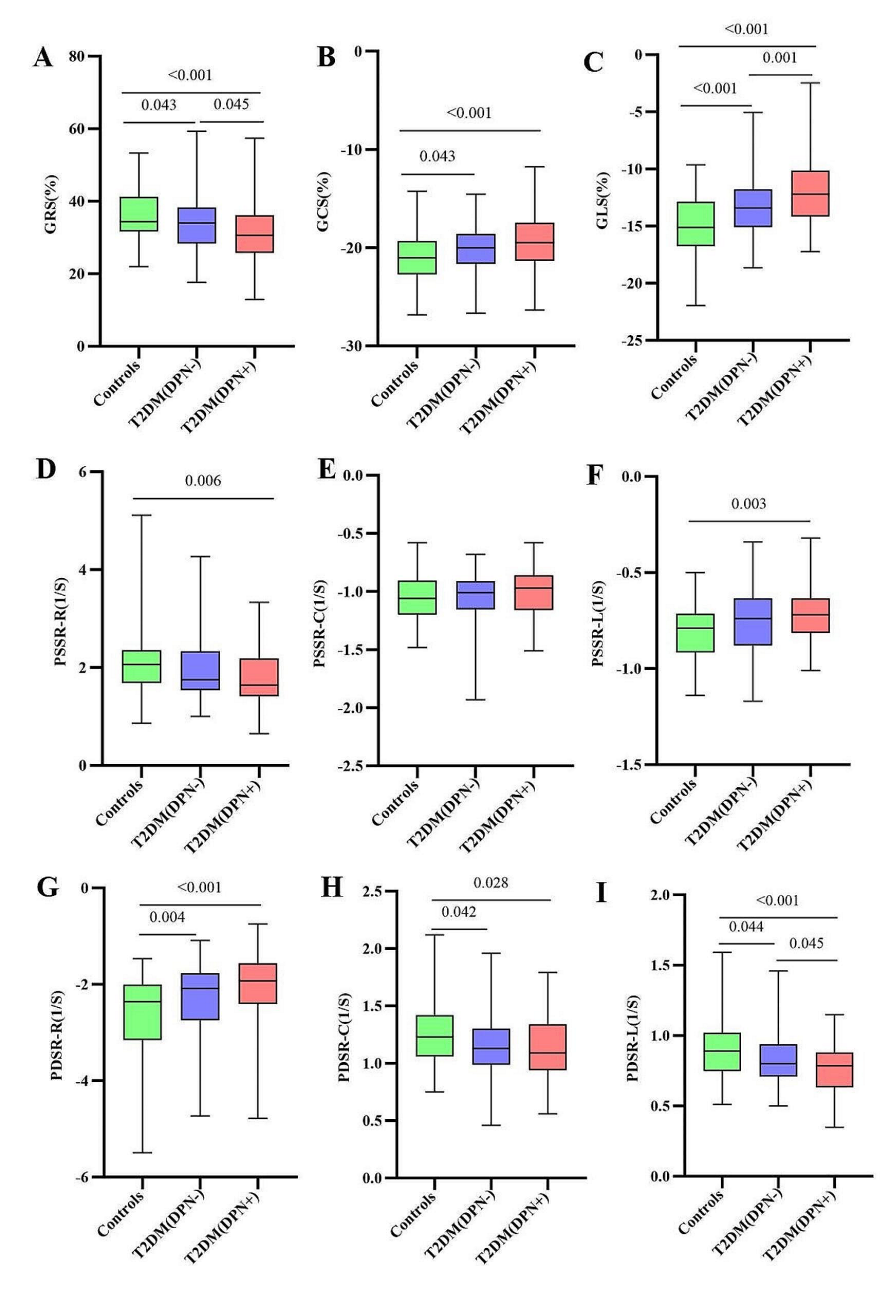
Comparion of left ventricular global strain parameters among controls, T2DM (DPN−) and T2DM (DPN+) groups. *GRS* global radial peak strain, *GCS* global circumferential peak strain, *GLS* global longitudinal peak strain, *PSSR* peak systolic strain rate, *PDSR* peak diastolic strain rate, *R* radial, *C* circumferential, *L* longitudinal, *T2DM* type 2 diabetes mellitus, *DPN* diabetic peripheral neuropathy

Regarding the strain parameters (Fig. 2), the LV radial and longitudinal PS, as well as longitudinal PDSR were decreased progressively from controls through T2DM patients without DPN to patients with DPN (all p < 0.001). LV radial and circumferential PDSR, as well as circumferential PS were reduced in both patient groups (all p < 0.05), but were not significantly different between these groups (all p > 0.05). In addition, radial and longitudinal PSSR were decreased in patients with DPN (p = 0.006 and 0.003, respectively) but preserved in patients without DPN (all p > 0.05).

### Determinants of subclinical LV dysfunction in T2DM patients

After univariable linear regression analyses (Tables 3 and 4), DPN was significantly associated with all three directions of LV global PS (all p < 0.05), radial and longitudinal PSSR and PDSR (all p < 0.1). Retinopathy was significantly associated with radial and longitudinal PS and PDSR, as well as radial and circumferential PSSR (all p < 0.05). Dyslipidemia was significantly associated with radial PS, PSSR and PDSR (all p < 0.05). In addition, LGE was significantly associated with all directions of LV global PS (all p ≤ 0.001), PSSR (p < 0.05) and PDSR (p < 0.1) except circumferential PSSR (p = 0.655). Results of other univariable analyses are shown in the tables.

**Table 3:** Univariable and multivariable analysis between the magnitude of LV peak strain and clinical indices in T2DM patients

	LV PS-longitudinal				LV PS-circumferential				LV PS-radial			
	Univariable		Multivariable		Univariable		Multivariable		Univariable		Multivariable	
	B	p	B^a^	p	B	p	B^a^	p	B	p	B^a^	p
DPN	1.592	< 0.001	1.187	0.003	0.894	0.027	0.105	0.149	− 2.818	0.019	− 3.030	0.014
Retinopathy	1.559	0.009	0.094	0.176	0.915	0.111			− 3.499	0.041	− 0.090	0.206
CKD	1.406	0.009	1.045	0.042	0.628	0.225			− 1.214	0.433		
Dyslipidemia	− 0.061	0.879			− 0.431	0.258			2.798	0.013	3.773	0.001
Hypertension	0.472	0.236			0.091	0.812			− 0.911	0.424		
Age (years)	− 0.007	0.744	− 0.046	0.492	− 0.02	0.308	− 0.030	0.098	0.011	0.857	0.111	0.119
Sex (male = 1)	1.816	< 0.001	1.691	< 0.001	1.446	< 0.001	1.168	0.003	− 3.546	0.002	− 2.970	0.01
BMI (kg/m^2)^	0.055	0.347			0.043	0.432			0.073	0.661		
HR (beats/min)	− 0.004	0.827			− 0.002	0.895			0.060	0.228		
Smoking	1.246	0.005	0.034	0.648	1.213	0.004	0.062	0.452	− 3.980	0.002	− 0.120	0.238
Diabetic duration (long = 1)	0.959	0.02	0.053	0.446	0.142	0.718	0.024	0.748	− 0.733	0.538	0.040	0.58
HbA1c (%)	0.138	0.193			0..151	0.133			− 0.414	0.15		
LV mass (g)	0.043	< 0.001	0.103	0.218	0.032	< 0.001	0.110	0.202	− 0.078	0.001	− 0.100	0.25
LGE	2.522	< 0.001	2.020	< 0.001	1.488	0.001	1.401	0.002	− 0.078	0.001	− 4.070	0.002
Triglycerides (mmol/L)	0.257	0.195			0.159	0.399			− 0.671	0.236		
Total cholesterol (mmol/L)	− 0.144	0.431			0.004	0.982			0.099	0.85		
HDL (mmol/L)	− 1.708	0.001	− 0.087	0.223	− 0.394	0.433			0.670	0.658		
LDL (mmol/L)	− 0.072	0.765			− 0.175	0.439			0.785	0.250		
Statin	0.086	0.846			− 0.005	0.991			− 0.474	0.707		
Biguanides Sulfonylureas	0.494	0.222			0.261	0.501			− 1.544	0.182		
α‑Glucosidase inhibitor	− 0.561	0.171			0.022	0.956			0.034	0.977		
GLP‑1/DDP‑4 inhibitor	− 0.030	0.963			− 0.668	0.273			2.516	0.167		
SGLT2	2.645	0.012	0.038	0.583	0.778	0.444			− 1.329	0.662		
Insulin	0.300	0.477			− 0.030	0.942			0.067	0.956		
ACEI/ARB	1.001	0.032	0.074	0.303	0.206	0.647			− 1.624	0.226		
β‑Blocker	0.943	0.178			0.612	0.361			− 1.362	0.498		
Calcium channel blocker	0.197	0.660			0.120	0.779			− 1.142	0.373		

Abbreviations as in Tables 1 and 2

^a^Variables with P < 0.1 in the univariable analysis as well as diabetic duration, age and sex were included in the multivariable analysis

**Table 4:** Univariable and multivariable analysis between the magnitude of LV peak systolic or diastolic strain rate and clinical indices in T2DM patients

	LV PSSR-longitudinal				LV PSSR-circumferential				LV PSSR-radial			
	Univariable		Multivariable		Univariable		Multivariable		Univariable		Multivariable	
	B	p	B^a^	p	B	p	B^a^	p	B	p	B^a^	p
DPN	0.050	0.054	0.096	0.213	0.050	0.108			− 0.202	0.045	− 0.266	0.007
Retinopathy	0.039	0.292			0.088	0.048	0.082	0.257	− 0.293	0.040	− 0.094	0.217
CKD	− 0.012	0.712			− 0.054	0.177			0.035	0.786		
Dyslipidemia	− 0.026	0.285			− 0.046	0.12			0.246	0.009	0.212	0.025
Hypertension	− 0.017	0.484			− 0.037	0.21			− 0.107	0.262		
Age (years)	0.001	0.764	− 0.003	0.018	0.001	0.843	− 0.003	0.051	− 0.005	0.282	0.072	0.337
Sex (male = 1)	0.002	0.947	0.0230	0.757	− 0.033	0.278	0.004	0.950	0.089	0.356	− 0.004	0.955
BMI (kg/m^2)^	0.002	0.508			− 0.001	0.862			0.014	0.299		
HR (beats/min)	− 0.006	< 0.001	− 0.007	< 0.001	− 0.008	0.001	− 0.009	< 0.001	0.018	< 0.001	0.020	< 0.001
Smoking	0.010	0.716			− 0.007	0.833			− 0.078	0.466		
Diabetic duration (long = 1)	0.054	0.003	0.070	0.006	0.024	0.432	0.063	0.028	− 0.13	0.175	− 0.097	0.193
HbA1c (%)	0.012	0.053	0.011	0.078	0.007	0.376			− 0.032	0.191		
LV mass (g)	0.001	0.619			− 0.001	0.197			0.001	0.971		
LGE	0.067	0.016	0.103	< 0.001	0.015	0.655			− 0.219	0.045	− 0.266	0.012
Triglycerides (mmol/L)	0.008	0.505			− 0.007	0.621			0.001	0.978		
Total cholesterol (mmol/L)	0.012	0.296			0.023	0.084	0.096	0.178	− 0.052	0.234		
HDL (mmol/L)	0.012	0.715			0.068	0.08	0.041	0.560	− 0.286	0.021	− 0.201	0.083
LDL (mmol/L)	0.016	0.275			0.028	0.114			− 0.01	0.865		
Statin	− 0.016	0.545			− 0.022	0.504			0.026	0.801		
Biguanides sulfonylureas	− 0.013	0.610			0.002	0.940			− 0.033	0.733		
α‑Glucosidase inhibitor	− 0.004	0.859			0.013	0.657			0.031	0.755		
GLP‑1/DDP‑4 inhibitor	− 0.011	0.786			− 0.006	0.896			0.197	0.193		
SGLT2	0.069	0.287			0.008	0.923			− 0.165	0.515		
Insulin	0.002	0.952			− 0.005	0.885			− 0.006	0.955		
ACEI/ARB	− 0.005	0.849			− 0.059	0.089	− 0.056	0.432	− 0.039	0.728		
β‑Blocker	− 0.005	0.903			0.002	0.676			− 0.052	0.755		
Calcium channel blocker	− 0.003	0.920			− 0.022	0.515			− 0.171	0.108		
DPN	− 0.080	0.008	− 0.086	0.001	− 0.025	0.512			0.205	0.077	0.281	0.016
Retinopathy	− 0.085	0.048	− 0.045	0.518	− 0.057	0.291			0.387	0.018	0.090	0.237
CKD	− 0.033	0.391			0.060	0.218			0.066	0.655		
Dyslipidemia	0.020	0.484			0.065	0.072	0.600	0.092	− 0.339	0.002	− 0.450	< 0.001
Hypertension	− 0.023	0.422			− 0.031	0.392			0.159	0.145		
Age (years)	0.002	0.761	0.003	0.049	− 0.002	0.277	− 0.030	0.682	0.009	0.088	0.086	0.235
Sex (male = 1)	− 0.072	0.013	− 0.102	0.18	− 0.080	0.028	− 0.070	0.068	0.287	0.009	− 0.107	0.144
BMI (kg/m^2)^	− 0.001	0.779			− 0.008	0.113			− 0.004	0.809		
HR (beats/min)	0.007	< 0.001	0.007	< 0.001	0.005	0.001	0.005	0.003	− 0.009	0.063	− 0.107	0.144
Smoking	− 0.071	0.026	− 0.061	0.391	− 0.026	0.526			0.251	0.039	0.084	0.265
Diabetic duration (long = 1)	− 0.018	0.506	− 0.102	0.18	− 0.013	0.724	− 0.020	0.763	0.056	0.611	− 0.046	0.542
HbA1c (%)	− 0.012	0.116			− 0.007	0.468			0.010	0.717		
LV mass (g)	− 0.002	0.001	− 0.002	0.006	− 0.002	0.007	− 0.001	0.091	0.007	0.001	0.006	0.004
LGE	− 0.085	0.009	− 0.076	0.014	− 0.072	0.081	− 0.080	0.318	0.330	0.008	0.074	0.355
Triglycerides (mmol/L)	− 0.021	0.132			− 0.022	0.224			0.037	0.495		
Total cholesterol (mmol/L)	0.014	0.295			0.001	0.991			− 0.032	0.527		
HDL (mmol/L)	0.046	0.214			0.042	0.372			− 0.023	0.873		
LDL (mmol/L)	0.018	0.281			0.006	0.775			− 0.084	0.200		
Statin	− 0.028	0.370			− 0.002	0.959			0.033	0.783		
Biguanides Sulfonylureas	− 0.020	0.494			− 0.007	0.842			0.205	0.064	0.198	0.070
α‑Glucosidase inhibitor	0.009	0.767			− 0.017	0.637			0.110	0.326		
GLP‑1/DDP‑4 inhibitor	0.021	0.638			0.065	0.260			− 0.201	0.249		
SGLT2	− 0.112	0.139			− 0.140	0.142			0.114	0.695		
Insulin	− 0.044	0.144			− 0.003	0.939			0.011	0.922		
ACEI/ARB	0.001	0.975			− 0.008	0.847			0.169	0.190		
β‑Blocker	− 0.051	0.310			− 0.099	0.119			0.305	0.111		
Calcium channel blocker	− 0.017	0.587			− 0.010	0.800			0.136	0.266		

Abbreviations as in Tables 1 and 2

^a^Variables with P < 0.1 in the univariable analysis as well as diabetic duration, age and sex were included in the multivariable analysis

Multivariable linear regression analyses adjusting for confounders demonstrated that DPN was independently associated with LV radial and longitudinal PS (β = − 3.030 and 1.187, p = 0.014 and 0.003, respectively) and PDSR (β = 0.281 and − 0.086, p = 0.016 and 0.001, respectively), as well as radial PSSR (β = − 0.266, p = 0.007). CKD was independently associated with LV longitudinal PS (β = 1.045, p = 0.042). Dyslipidemia was independently associated radial PS, PSSR and PDSR (β = 3.773, 0.212 and -0.450, all p < 0.05). Additionally, LGE was independently associated with radial, circumferential and longitudinal PS (β = − 4.070, 1.401 and 2.020, all p ≤ 0.002), radial and longitudinal PSSR (β = − 0.266 and 0.103, p = 0.012 and < 0.001, respectively), and longitudinal PDSR (β = − 0.076, P = 0.014).

## Discussion

Assessment of myocardial deformation by strain and strain rate is sensitive to detect subclinical myocardial systolic and diastolic dysfunction, in which PS and PSSR reflect myocardial systolic function, while PDSR is a sensitive marker of LV diastolic dysfunction. In T2DM patients without complicated DPN, we observed increases in LV myocardial mass and remodeling index and decreases in three directional PS and PDSR compared with the controls, but no significant differences in LVEDVI, LVESVI, LVSVI and LVEF among the groups. These findings indicate that LV myocardial systolic and diastolic function measured by CMR-FT were impaired at the early stage before the reduction of LVEF, which was consistent with previous studies [14, 15]. In addition, the presence of LGE indicating myocardial fibrosis was also observed in these patients. Taken together, detection of early alternations in the LV myocardium enable early intervention and implementation of preventative strategies in T2DM patients.

In diabetes, hyperglycemia and lipotoxicity related to insulin resistance may lead to suppressed glucose oxidation, increased free fatty acid metabolism, inadequate calcium handling, mitochondrial dysfunction, increased oxidative stress, interstitial and perivascular fibrosis, and cardiomyocyte hypertrophy and stiffness, which may contribute to reduced ventricular compliance at the early stage [16, 17]. A recent meta-analysis including a large number of patients (n = 5053) showed that diabetes was associated with a higher degree of myocardial fibrosis assessed by histological collagen volume fraction and extracellular volume fraction [18], and previous studies have detected diastolic dysfunction in the diabetic hearts without hypertrophy [19, 20]. With the aggravation of aforementioned pathologies along with impairment in excitation–contraction coupling, microvascular abnormalities manifesting as microvascular endothelial inflammation, rarefaction and perivascular collagen, and end-product deposition, and increased LV wall thickness and mass may lead to systolic dysfunction [21, 22]. Some studies have demonstrated an adverse effect of T2DM on subclinical LV systolic strains and myocardial microvascular impairment [23, 24].

Further analysis in our patients revealed that the magnitude of LV radial and longitudinal PS as well as longitudinal PDSR were markedly lower in patients with DPN than in both controls and patients without DPN, and radial and longitudinal PSSR were reduced in patients with DPN but preserved in those without DPN. However, the LV geometry was not significantly different between the patient groups. In addition, we identified that DPN was independently associated with the magnitude of LV radial and longitudinal PS and PDSR, as well as radial PSSR after adjustment for confounding factors. Thus, we speculated that subclinical LV dysfunction was progressed in T2DM patients with DPN even without progressive alterations in LV geometry, which was consistent with previous speckle-tracking echocardiography study [25]. Subendocardial myocardial fibers predominantly affected by coronary microvascular dysfunction are impaired early and severer, then manifesting as independent association between DPN and LV longitudinal PS and PDSR. The results that DPN was not associated with circumferential PS and PDSR indicate subepicardial myocardial impairment was not decreased progressively in our patients with DPN. Because both subendocardial and subepicardial fibers contribute to LV radial function[26], decreased LV radial PS and PDSR may mainly be caused by impaired subendocardial fibers when circumferential function was not progressively decreased.

Several studies have shown that the main mechanisms involved in DPN are longstanding hyperglycemia, dyslipidemia and insulin resistance, which may cause common pathophysiological changes in multiple organs, such as mitochondrial dysfunction, oxidative stress, accumulation of advanced glycation end products, lipotoxicity, increased inflammatory cytokine synthesis and microvascular complications [3, 27, 28]. Myocardial dysfunction may be involved when these changes occur in the heart. An elevated HbA1c level is a known cardiovascular risk factor and associated with higher degrees of myocardial fibrosis [18], its reduction will lead to reduced risks of both macro- and microvascular disease [29]. Our results revealed higher HbA1c levels in patients with DPN, indicating poor glycemic control, higher metabolic disorder and myocardial fibrosis; however, it was not associated with myocardial dysfunction. A potential explanation for this discrepancy is that HbA1c levels may not be a good indicator of long-term glycemic control, as it only reflects glycemic control over the past 3–4 months. Besides, we found that patients with DPN had a higher incidence of dyslipidemia and it was independently associated with worsening LV radial PS, PSSR and PDSR, which may indicate that determining the pathophysiological mechanisms underlying the effect of dyslipidemia will provide mechanistic targets for developing new targeted therapies for DPN and related myocardial dysfunction.

A previous study revealed that microvascular alterations, similar to those observed in diabetic retinopathy and nephropathy, appear to be associated with pathological alterations of nerves [30], which may lead to reduced peripheral nerve nutrition and impaired nerve function. Chung et al. reported a more frequent prevalence of retinopathy in patients with T2DM presenting peripheral neuropathy [31], and it was consistent with our findings. Reduced flow in the left anterior descending artery was observed in patients with retinopathy [32]. The study by Sørensen et al. reported a decrease in myocardial perfusion reserve in patients with retinopathy that was associated with diastolic dysfunction [33]. According to our results, the retinopathy and CKD were significantly associated with LV myocardial dysfunction. Zhang et al. demonstrated that kidney dysfunction may aggravate the deterioration of LV strain in T2DM patients [34], and another study revealed that the LV global longitudinal strain is a superior predictor of all-cause and cardiovascular mortality when compared with ejection fraction in advanced CKD patients [35]. In addition, the study by Baltzis et al. showed that patients with DPN had a higher risk of myocardial ischemia than those without DPN using technetium-99 m sestamibi single-photon emission computed tomographic imaging [36], which was consistent with our finding that patients with DPN had higher proportion of non-infarct LGE indicating severer microvascular dsyfunction [37]. These observations suggest that the common mechanism of microvascular impairment in diabetic complications plays an important role in myocardial dysfunction in patients with DPN. It is highly stimulating developing future targeted medications improving microvascular function to improve prognosis in T2DM patients; for example, glucagon-like peptide-1(GLP-1) has been shown to have benefits for patients with microvascular complication [38].

LGE could be used as a surrogate for replacement fibrosis and very little is known regarding nonischemic LGE implications in T2DM patients [39]. In the present study, we found a high prevalence of non-infarct LGE in T2DM patients and even higher proportion in those with DPN, and it was independently associated with worsening LV systolic (three directional PS as well as radial ad longitudinal PSSR) and diastolic (longitudinal PDSR) dysfunction. A previous study showed that T2DM patients with non-ischemic LGE lesions had increased ECV [39], and a recent study revealed that coronary microvascular dysfunction was significantly associated with the development of myocardial fibrosis in patients with T2DM [40]. Considering higher prevalence of non-infarct LGE in patients with DPN, they may have severer myocardial structural and functional impairment in T2DM with DPN, which may explain the poor cardiac outcomes in these patients.

## Limitations

There are several limitations in our study. Firstly, this was a retrospective, single-center, observational study involving a relatively limited sample size, which may introduce selection bias and limit the ability to establish causality. Therefore, a prospective, multicenter study is desirable to validate our results. Secondly, we only included diabetic patients without heart failure and preserved LVEF, the generalizability of our findings to patients with heart failure is worth to be further investigated. Thirdly, Although LGE is the technique of choice for diagnosis of replacement fibrosis, it cannot evaluate diffuse myocardial fibrosis. Native and postcontrast T1-mapping can assess the extent and distribution of diffuse myocardial fibrosis, however, it was not available in our series and will be implemented in our further studies. Furthermore, not all the patients underwent nerve conduction tests, and selection bias may exist because subclinical DPN with no signs or symptoms of neuropathy could not be diagnosed. However, our data reflect routine clinical practice in diagnosing DPN, and further studies are required to explore LV changes in patients with subclinical DPN. Finally, the inherent cross-sectional design of this study prevented us from drawing conclusions on causality. Long-term longitudinal studies are needed to investigate the ability of impaired LV strains and non-infarct LGE to predict cardiovascular outcomes in patients complicated with DPN.

## Conclusions

There was more non-infarct LGE lesions and worsening subclinical LV dysfunction in T2DM patients complicated with DPN than those without DPN, which may suggest further prospective study with even more extensive therapeutic interventions in this cohort of patients to improve patient outcomes.

## Data Availability

The datasets used and analyzed during the current study are available from the corresponding author on reasonable request.

## Abbreviations

T2DM: Type to diabetes mellitus
DPN: Diabetic peripheral neuropathy
LV: Left ventricular
CMR: Cardiovascular magnetic resonance
CMR-FT: CMR feature tracking
EDV: End-diastolic volume
ESV: End-diastolic volume
SV: Stroke volume
CO: Cardiac output
EF: Ejection fraction
LVMI: LV mass index
PS: Peak strain
PSSR: Peak systolic strain rates
PDSR: Peak diastolic strain rates
LGE: Late gadolinium enhancement
